# Contrasting Effects of Intraspecific Trait Variation on Trait-Based Niches and Performance of Legumes in Plant Mixtures

**DOI:** 10.1371/journal.pone.0119786

**Published:** 2015-03-17

**Authors:** Christiane Roscher, Jens Schumacher, Bernhard Schmid, Ernst-Detlef Schulze

**Affiliations:** 1 UFZ, Helmholtz Centre for Environmental Research, Department of Community Ecology, Halle, Germany; 2 Institute of Stochastics, Friedrich Schiller University, Jena, Germany; 3 Institute of Evolutionary Biology and Environmental Studies and Zurich-Basel Plant Science Center, University of Zurich, Zurich, Switzerland; 4 Max Planck Institute for Biogeochemistry, Jena, Germany; Università Politecnica delle Marche, ITALY

## Abstract

Niche differentiation, assumed to be a key mechanism of species coexistence, requires that species differ in their functional traits. So far it remains unclear to which extent trait plasticity leads to niche shifts of species at higher plant diversity, thereby increasing or decreasing niche overlap between species. To analyse this question it is convenient to measure niches indirectly via the variation in resource-uptake traits rather than directly via the resources used. We provisionally call these indirectly measured niches trait-based niches. We studied shoot- and leaf-morphological characteristics in seven legume species in monoculture and multi-species mixture in experimental grassland. Legume species varied in the extent of trait variation in response to plant diversity. Trait plasticity led to significant shifts in species niches in multiple dimensions. Single-species niches in several traits associated with height growth and filling of canopy space were expanded, while other niche dimensions were compressed or did not change with plant diversity. Niche separation among legumes decreased in dimensions related to height growth and space filling, but increased in dimensions related to leaf size and morphology. The total extent of occupied niche space was larger in mixture than in the combined monocultures for dimensions related to leaf morphology and smaller for dimensions related to whole-plant architecture. Taller growth, greater space filling and greater plasticity in shoot height were positively, while larger values and greater plasticity in specific leaf area were negatively related with increased performance of species in mixture. Our study shows that trait variation in response to plant diversity shifts species niches along trait axes. Plastically increased niche differentiation is restricted to niche dimensions that are apparently not related to size-dependent differences between species, but functional equivalence (convergence in height growth) rather than complementarity (divergence in traits associated with light acquisition) explains increased performance of legumes in mixture.

## Introduction

Niche differentiation among species has been identified as potential key promoter of species coexistence and biodiversity [1–5]. A major assumption of the niche-based theory is that decreasing levels of niche overlap, i.e. greater differences in niche means between species and smaller amounts of species`niche widths, are a prerequisite for species coexistence [6]. The classical niche-based theory formulates niche differences among species as the ability to exploit different environments, which may be expressed as changes in species performance. However, to measure niches it can be more convenient to use an indirect method, namely to measure variation in resource-uptake traits instead of measuring variation in the resources used [2,3,7]. Here we provisionally call these indirectly measured niches trait-based niches. A similar concept has recently been suggested in the literature and also relies on the possibility that niches may be quantified based on functional traits [8,9], where trait dissimilarity is assumed to decrease niche overlap among co-existing species. In this framework, plant functional traits are defined as morphological, physiological and phenological characteristics of individuals that are directly or indirectly related to plant performance [10]. Plant individuals may plastically adjust their trait values in response to abiotic or biotic conditions. Intraspecific trait variation may change the realized niche of individual species, but has been mostly neglected as a possible mechanism explaining increased complementarity at higher plant diversity.

Plasticity in trait expression depends on genetic, environmental, developmental and stochastic factors [2,3,11–13] and is fundamental for plants to cope successfully with changing environmental conditions [14]. Different traits often co-vary within plant individuals. The need to coordinate different functions to maximize performance and to respond to varying environmental conditions may constrain independent plastic responses of single traits [13,15] resulting in correlations among functionally related traits (phenotypic integration [16,17]).

At the level of plant communities, differential plastic responses of species to environmental conditions may alter the strength of interspecific competition. Not all trait plasticity, however, would increase the performance of the partners involved in it. In particular, trait plasticity in response to variation in light availability may be difficult because of the asymmetric nature of competition for light, i.e. taller individuals shading out smaller ones without reciprocal effects [18,19]. Thus, plasticity in size-related traits, such as plant height, could be expected to increase similarity among species, i.e. trait convergence, when species with different growth stature are grown in mixture as opposed to monoculture. Such size-related plastic responses to vegetation shade, helping all individuals to reach as much light as possible, include shoot and leaf elongation, a larger investment into supporting tissue and increased apical dominance with reduced branching [20,21]. However, once these possibilities have been used up and a species is faced with reduced light availability under the shade of taller competitors, it still has the potential for plastic responses in other traits of light acquisition, e.g. the formation of leaves with a larger specific leaf area, altered leaf orientation or even shifting its light absorption spectrum [22,23].

In temperate grasslands high plant species diversity usually results from the coexistence of a large number of subordinate species with a few tall-stature dominant species [24]. Grassland biodiversity experiments have shown that community biomass production and canopy density is larger in multi-species communities than in monocultures [25–27]. Spatial partitioning of light due to interspecific differences in structural and physiological characteristics, which allow species to use different positions in vertical space and increase resource exploitation in terms of light interception, have been used to explain the coexistence of many species in productive grasslands [28], while less is known about the role of intraspecific trait variation for species performance and coexistence.

Here, we present results of a study carried out in the Jena Experiment [29]. This grassland biodiversity experiment comprises monocultures and mixtures of different species richness and composition based on a pool of 60 temperate grassland species. Traits related to light acquisition and describing height growth, space filling and leaf morphology of seven legume species were measured using only monocultures and plots of maximum diversity, which are 60 sown species. The choice of legume species as a target group for our study was motivated by their unique ability to fix atmospheric N_2_. Legumes are therefore often considered as a plant functional group that is independent of competitive nitrogen supply from soil, but legume species differ considerably in their height growth and shoot morphology. In addition, results from biodiversity experiments including studies on functional trait variation of non-legume species (e.g. [30,31]) have shown strong effects of legume presence-absence, which often cannot be disentangled from species-richness effects because of an increasing chance to include legumes at higher levels of plant diversity. Thus, our focus on legume species allows for a separation of species-richness effects from legume effects because all studied communities (even the monocultures) contained legumes.

**Fig 1:**
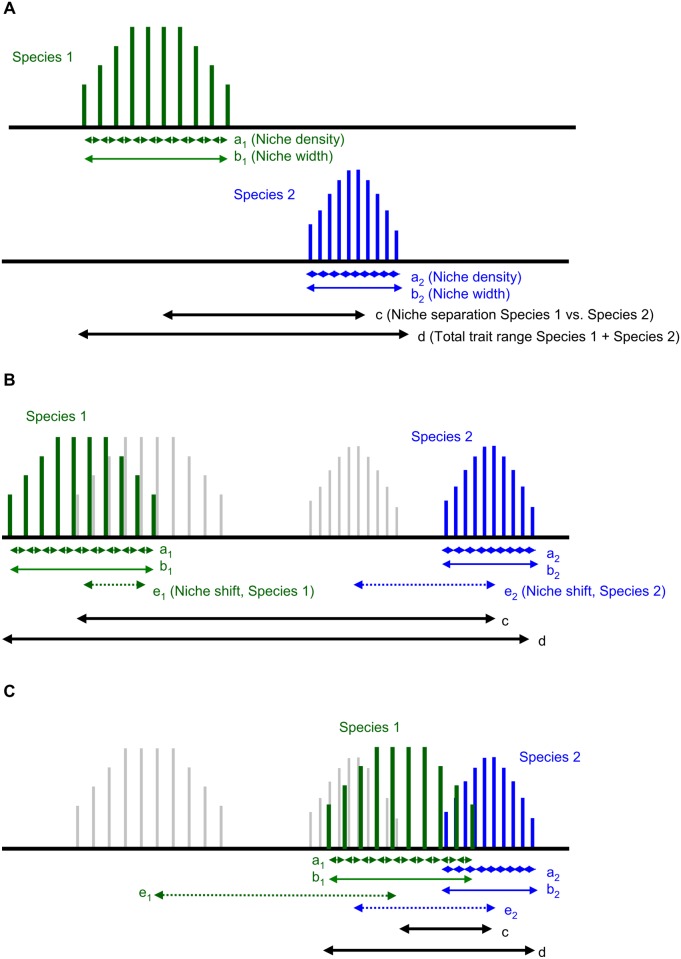
Quantification of species niches along a trait axis. For simplicity only two species (Species 1, Species 2) are considered in monoculture (A) and in a scenario for (B) trait divergence and (C) trait convergence in mixture. Arrows indicate the extent of niche density (a_1_, a_2_) and niche width (b_1_, b_2_) for each species in the monoculture and the mixture, niche separation between Species 1 and Species 2 (c), the total trait range across both species (d), and niche shift (e_1_, e_2_) of each species in the mixture compared to the monoculture. Grey—shaded lines in (B, C) visualize the position of Species 1 and Species 2 respectively in monoculture.

First, we analysed how variable are traits related to light acquisition in response to increased plant diversity in the studied legume species. Second, we asked whether plant diversity affects phenotypic integration. We expected that a species’ phenotypic integration increases from monoculture to mixture because plastic adjustments to optimize light capture and photosynthetic carbon gain must be coordinated within plants. Third, we wanted to test whether trait plasticity, i.e. a shift of species trait values in plant mixture compared to the monoculture, affects legume species`niche position and niche width, niche separation among legume species and the trait range of multiple legume species taken together as a group (Fig. 1).

We hypothesized that species diverge in traits related to space filling and leaf morphology (Fig. 1B) and converge in traits related to height growth (Fig. 1C) in the mixture. Fourth, we evaluated the relationships between diversity effects on species performance in terms of shoot biomass and trait-based niches to test the hypothesis that trait variation decreases niche overlap and increases complementarity among legume species in the mixture.

## Material and Methods

### Study site and experimental design

The Jena Experiment was established on a formerly highly fertilized agricultural field in 2002. The field site was rented by the research consortium of the Jena Experiment from an agricultural collective. No specific permission was required for the described study. The Jena Experiment field is not subject to protection for nature conservation and the study did not involve endangered or protected species. The experimental site is located in the floodplain of the river Saale near to the city of Jena (Thuringia, Germany, 50°55`N, 11°35`E, 130 m a.s.l.). The area has a mean annual air temperature of 9.3°C and annual precipitation amounts 587 mm [32]. The soil is a Eutric Fluvisol and soil texture changes from sandy loam near the river to silty loam with increasing distance to the river.

A detailed description of the experiment is given in [29]. Briefly, sixty experimental species typically occurring in Central European semi-natural mesophilic grasslands (Molinio-Arrhenatheretea [33]) were chosen and classified into four functional groups: 16 grasses, 12 small herbs, 20 tall herbs, and 12 legumes. Apart from 78 plots of 20 × 20 m size with plant communities of different species richness (1, 2, 4, 8, and 16) randomly assembled from species representing a different number of functional groups (1, 2, 3, and 4), mixtures with all 60 experimental species at equal proportions were sown on four large plots. In addition, each species was grown in monoculture on smaller plots of 3.5 × 3.5 m size. The experimental plots were arranged in four blocks each containing an equal number of plots per diversity level to account for the systematic variation in soil characteristics. The study presented here used the replicated 60-species mixtures and a monoculture of each investigated species (see below). The field site was not fertilized during the experiment. Plots were mown twice each year (June, September) and mown biomass was removed. To maintain sown species combinations all plots were weeded yearly in April and July when the vegetation was low and the canopy not completely closed.

### Data collection

Seven legume species, which were well established in their monoculture as well as in the 60-species mixture, were investigated in the present study. Our study was restricted to these diversity levels because the random allocation of species to the mixtures in the Jena Experiment does not afford that individual species are equally represented at intermediate species-richness levels, which was an important prerequisite for our analyses. The chosen legume species were *Lathyrus pratensis* L., *Lotus corniculatus* L., *Medicago x varia* Martyn, *Onobrychis viciifolia* Scop., *Trifolium hybridum* L., *T*. *pratense* L. and *Vicia cracca* L. The clonal growth with belowground stolons in two species (*L*. *pratensis*, *V*. *cracca*) impedes a clear separation of genetic individuals (genets) in the field. Therefore, single shoots served as basic unit for all measurements.

In late May 2005, 3 years after sowing, one or several transects perpendicular to the plot margin excluding the outer 70 cm of the plot were installed in monoculture and in two of the replicated 60-species mixture plots. The large plots (20 × 20 m plots) were used for sampling whenever possible. Four legume species, which were not grown on large monoculture plots had to be sampled on the small plots (3.5 × 3.5 m; *L*. *corniculatus*, *T*. *hybridum*, *T*. *pratense*, *V*. *cracca*), but previous analyses of plant productivity did not identify plot-size effects in the Jena Experiment [34]. Along transects one shoot per species was sampled every 25 cm choosing the shoot rooting closest to the sampling point. Because light is exponentially attenuated towards the ground in closed stands of vegetation [35], shoot height and canopy height of the surrounding vegetation were measured to assess the positioning of individual shoots in the canopy. Three vertical segments were defined for each shoot according to their position in the surrounding vegetation (Q1: 0–25% height, Q2: 25–75% height, Q3: >75% height), i.e. smaller shoots often did not reach the uppermost category. Angles of shoot axes and leaf blades were estimated using a scale with six categories (0°–30°, 31°–60°, 61°–90°, 91°–120°, 121°–150°, 151°–180° deviation from an ascending vertical) in each segment. Afterwards, shoots were cut off at ground level, put in sealed plastic bags and transported in a cool box to the laboratory. In total, 9–12 shoots per species were harvested in the monoculture and the 60-species mixture respectively. In the laboratory, stretched shoot length and the lengths of three to five internodes in the central part of the shoot axis were measured and the number of secondary axes was counted. Then, shoots were cut into segments (as marked in the field). Plant material was separated into compartments (supporting parts = stems and higher-order axes, leaves, reproductive parts = inflorescences and fruits). One to twelve (dependent on leaf size, number and availability) fully expanded leaves from each vertical segment were scanned with a flatbed scanner (Epson Perfection, 4180P). Scanned leaves were analysed with image software (Gimp2) to derive leaf area and length of the leaf blade. All plant fractions were oven-dried at 70°C (48 h) and weighed. Variables derived from these measurements for further analyses are defined in Table 1.

**Table 1:** Overview of traits related to light acquisition analysed in this study.

Variable	Unit	Description
**Height growth**		
Shoot height	cm	growth height of a shoot measured in the field
Shoot length	cm	stretched shoot length measured in the laboratory
Internode length	cm	length of the longest internode per shoot
Stem mass fraction	mg_stem_ mg^-1^_shoot_	stem dry mass (including secondary axes) per shoot dry mass = SMF
**Space filling**		
Stem angle basal	°	stem angle at the shoot base (Q1: 0–25% height)
Stem angle	°	stem angle in the canopy (Q2-Q3: 25–100% height)
No. secondary axes		number of secondary and higher order lateral axes per shoot
Leaf number		number of leaves per shoot
Leaf angle max*	°	angle of leaf blades (maximum deviation from an ascending vertical in Q1-Q3)
Leaf angle min*	°	angle of leaf blades (minimum deviation from an ascending vertical in Q1-Q3)
**Leaf morphology**		
Leaf length	mm	length of the longest leaf
Leaf area	cm^2^	area of the largest leaf
Specific leaf area max**	mm^2^_leaf_ mg^-1^_leaf_	leaf area per leaf dry mass (maximum values) = SLA_max_
Specific leaf area min**	mm^2^_leaf_ mg^-1^_leaf_	leaf area per leaf dry mass (minimum values) = SLA_min_
**Performance**		
Shoot biomass	g	biomass of shoots

* Maximum and minimum leaf angles were defined by using either maximum (largest deviation from an ascending vertical) or minimum (smallest deviation from an ascending vertical) values estimated as average per segment (Q1-Q3).

** Specific leaf area was introduced in analyses as maximum and minimum values measured in segment Q1, Q2 or Q3.

### Data analyses

Data were analysed with the statistical software R3.1.1 [36] including the *lmer* function in the package *lme4* [37].

Mixed-effects models were used to evaluate how variable traits were in response to increased plant diversity in the studied legume species (question 1). Block and plot identity were entered as random effects in a nested sequence. Starting from a constant null model, species identity, plant diversity (monoculture vs. mixture) and the interaction between both terms were added stepwise as fixed effects. The maximum likelihood method was applied and likelihood ratio (Chi^2^) tests were used to assess the statistical significance of adding terms to the model. If necessary, trait data (shoot length, internode length, number of secondary axes, leaf number, leaf length, leaf area, specific leaf area) were log-transformed to meet the assumptions of statistical analyses. Tukey`s tests applying the *glht* function in the package *multcomp* [38] in models fitted with the restricted maximum likelihood method (REML) were used to identify significant differences between trait values in monoculture and mixture populations of individual legume species. In addition, regression slopes against species richness (monoculture vs. mixture) were calculated to assess how the reaction norm in response to plant diversity varied among species and traits. Trait values were z-transformed (= standardized to mean = 0, variance = 1) to account for different scaling of trait data.

For all subsequent analyses trait data were corrected for block effects. Pearson correlation coefficients for all pair-wise trait combinations in monoculture and mixture populations of each legume species were computed using trait data of each shoot as a sample to test whether plant diversity affects phenotypic integration (question 2).

Euclidean distances based on standardized values of single and multiple traits were used to evaluate how plant diversity affects trait-based niches of legume species in monoculture and mixture (question 3). Mean values of all possible pair-wise within-species distances per diversity level were used to estimate *niche density* for each species, where small mean distances indicate a greater niche density. Maximum values of all possible pair-wise within-species distances per diversity level served to estimate total *niche width* for each species. Mean values of all possible pair-wise distances to other species per diversity-level were explored as measure of *niche separation*. Mean values of all pair-wise within-species distances of monoculture individuals to mixture individuals per species were compared to within-species distances of monoculture individuals as a measure of *niche shift* in response to increasing plant diversity. A paired t-test served to test for differences in total niche width between monoculture and mixture. A two-way ANOVA based on the factors species identity and plant diversity and their interaction was used to test for effects on niche density, niche separation and niche shift. Tukey`s test was applied for multiple pair-wise comparisons of means per species. Mean and maximum trait distances across individuals of all legume species were explored to evaluate the *filling of trait space* and the *trait range* of multiple species in monocultures and in mixture (Fig. 1). A methodological caveat that could not be prevented was that variation among species in monoculture always included variation among plots, because, for monocultures, plots and species identity were confounded. In contrast, variation among species in mixture did not include a variance component for between-plot differences. As a consequence, tests for increased differences among species in mixture than in monoculture were more conservative than tests the other way round.

To test whether trait variation decreases niche overlap and increases complementarity among legume species in the mixture (question 4), diversity effects on species performance in terms of shoot biomass were quantified as proportional deviation from expected values as

$$D=\frac{BM_{Mix}-\bar{BM_{Mono}}}{\bar{BM_{Mono}}}$$ (eqn. 1),

where *BM*_*Mix*_ is shoot biomass in the mixture, and $\bar{BM_{Mono}}$ is the mean shoot biomass of the respective species in monoculture [39]. Mixed-effects models were applied to test for relationships between diversity effects on shoot biomass and trait-based niches in the mixture. Plot and species identity were included as independent random effects to account for multiple observations per plot and per species. Starting with a model with species identity as fixed effect to take systematic differences between species into account, alternative predictor variables, i.e. within-species trait distances (niche density), trait distances to other species (niche separation), within-species distances to the monoculture (niche shift) or trait values (niche identity) of the respective shoot were tested separately. The Akaike Information Criterion (AIC) was used for between-model comparisons, and likelihood ratio tests were applied to test for significant model improvement by adding predictor variables.

## Results

### Extent of trait variation in response to plant diversity

**Fig 2:**
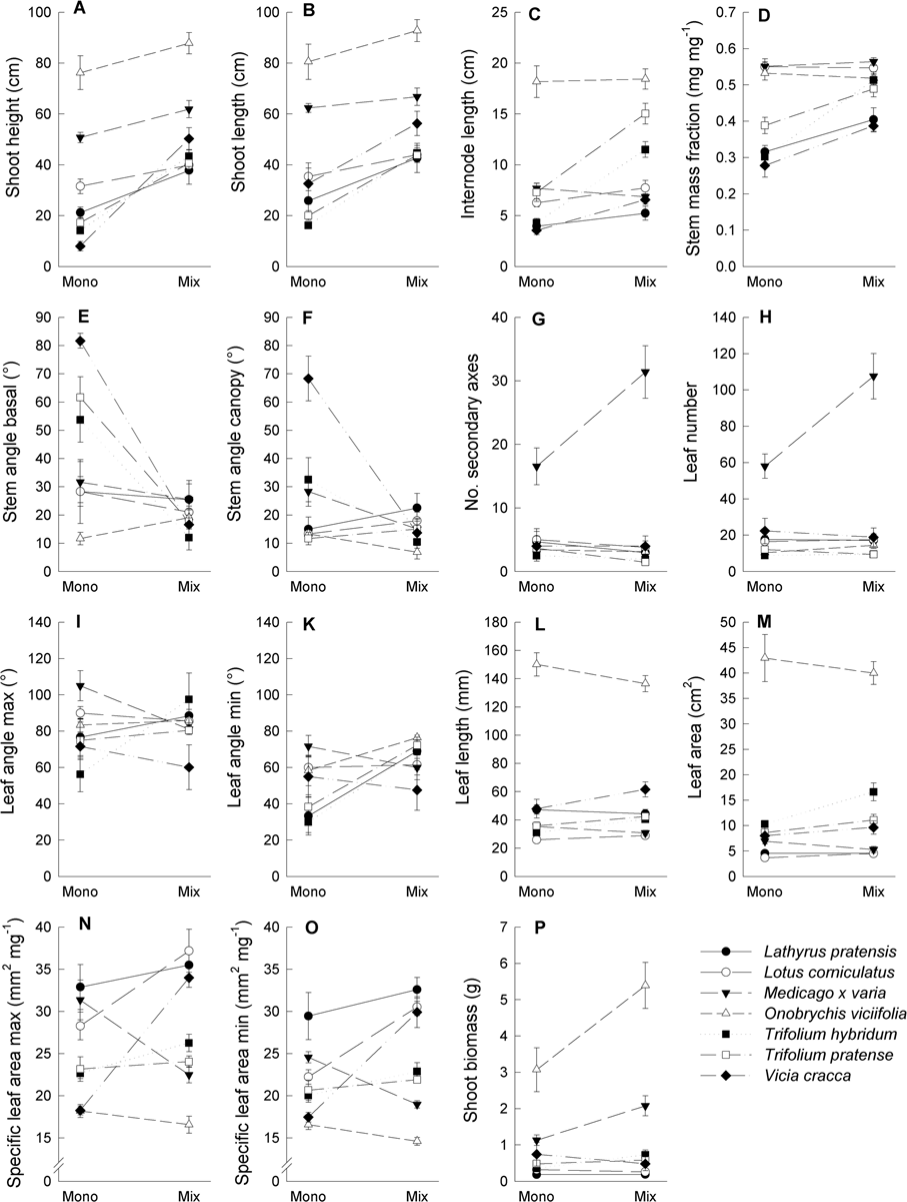
Traits related to light-acquisition and performance measured as shoot biomass of seven legume species studied in monoculture and mixture. Shown are mean values (±1 SE) per diversity level for (A) shoot height, (B) stretched shoot length, (C) internode length, (D) stem mass fraction, (E) stem angle at shoot base, (F) stem angle in the canopy, (G) number of secondary axes, (H) leaf number, (I) maximum leaf angle, (K) minimum leaf angle, (L) leaf length, (M) leaf area, (N) maximum specific leaf area, (O) minimum specific leaf area, and (P) shoot biomass.

Legume species differed in all investigated traits except for stem and leaf angles (Table 2; Fig. 2).

**Table 2:** Summary of mixed-effects models testing for effects of legume species identity and plant diversity on expression of traits related to light acquisition and performance in terms of shoot biomass.

Variable	Species	Diversity	Species x Div	Lp	Lc	Mv	Ov	Th	Tp	Vc
Shoot height	126.27***	6.35*↑	28.22***	—	—	—	—	***↑	***↑	***↑
Shoot length	62.96***	4.83*↑	28.51***	*↑	—	—	—	***↑	***↑	***↑
Internode length	127.64***	2.31	34.90***	—	—	—	—	***↑	***↑	***↑
Stem mass fraction	62.22***	2.33	30.86***	—	—	—	—	***↑	*↑	*↑
Stem angle basal	2.64	1.74	33.83***	—	—	—	—	***↓	***↓	***↓
Stem angle canopy	7.01	0.68	34.74***	—	—	—	—	*↓	—	***↓
No. secondary axes	71.59***	0.48	6.59	—	—	—	—	—	—	—
Leaf number	125.63***	1.97	8.47	—	—	—	—	—	—	—
Leaf angle max	10.37	0.31	21.69***	—	—	—	—	**↑	—	—
Leaf angle min	8.42	1.35	23.64***	—	—	—	—	—	—	—
Leaf length	203.11***	2.31	20.52**	—	—	—	—	—	—	—
Leaf area	189.01***	3.37	15.54*	—	—	—	—	—	—	—
Specific leaf area max	135.89***	0.29	39.48***	—	**↑	***↓	—	—	—	***↑
Specific leaf area min	145.04***	0.41	38.32***	—	***↑	**↓	—	—	—	***↑
Shoot biomass	140.97***	5.66*↑	0.28	—	—	—	—	—	—	—

Models were fitted by stepwise inclusion of fixed effects. The first columns show results of likelihood-ratio tests that were applied to assess model improvement (Chi^2^) and the statistical significance of the explanatory terms (Species = species identity, Diversity = monoculture vs. mixture), where

* P ≤ 0.05,

** P < 0.01,

*** P < 0.001.

Arrows indicate increases (↑) or decreases (↓) in trait values in mixture compared to monocultures. Tukey`s test was applied for multiple pair-wise comparisons of sample means per species. Abbreviations are Lp = *Lathyrus pratensis*, Lc = *Lotus corniculatus*, Mv = *Medicago x varia*, Ov = *Onobrychis viciifolia*, Th = *Trifolium hybridum*, Tp = *Trifolium pratense*, Vc = *Vicia cracca*.

Shoot height and stretched shoot length increased at higher plant diversity, while the response of other traits to increased plant diversity was more variable. Trait variation depended largely on species identity except for variation in the number of secondary axes and leaf number. Ranking of reaction norms (standardized regression slopes against plant diversity) provided further evidence that species varied considerably in the identity of traits that responded to plant diversity and in the degree to which they did so (S1 Fig.). While the majority of traits reached higher values in mixture than in monoculture (positive slopes with values > 0) in five species (*L*. *pratensis*, *O*. *viciifolia*, *T*. *hybridum*, *T*. *pratense*, *Vicia cracca*), the opposite was observed in the two remaining species where the majority of values was < 0 (*L*. *corniculatus*, *M*. *x varia*).

### Effects of plant diversity on phenotypic integration

The number of significant within-species trait correlations (P ≤ 0.05) as a measure of phenotypic integration ranged from 9–17 in monocultures and from 11–40 in mixture out of 91 possible correlations. It was higher in mixture than in monoculture for all species except *L*. *corniculatus* and *O*. *viciifolia*. The identity of traits with a high number of correlations differed largely between species and within species between monoculture and mixture (S2 Fig.).

### Niche positioning of single species along trait axes in monoculture and mixture

**Fig 3:**
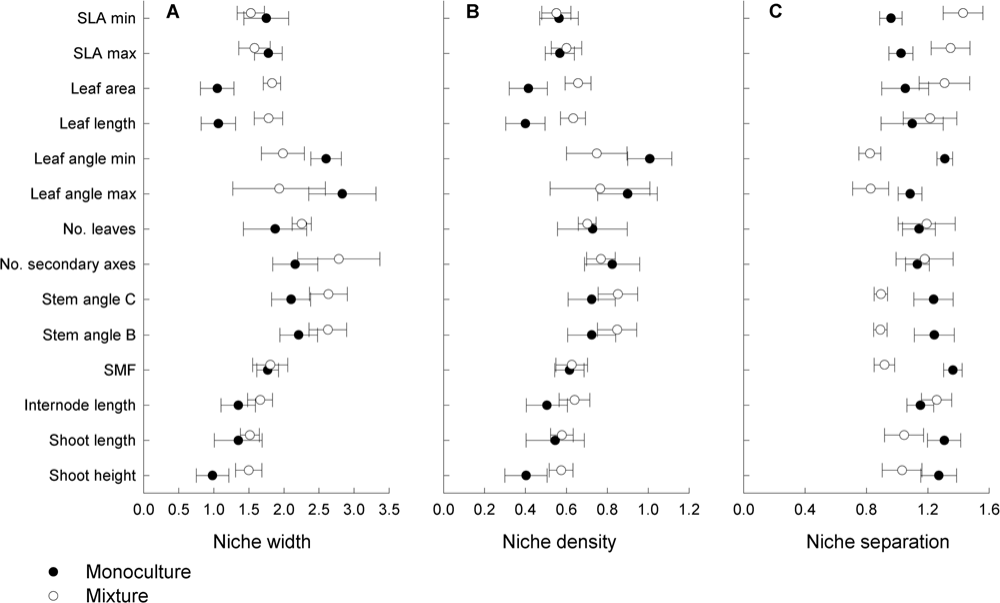
Niche width, niche density and niche separation of legume species in monoculture and mixture. Niche width was estimated as within-species maximum trait distance (A), niche density was estimated as within-species mean trait distances, i.e. smaller distances = greater niche density (B), and niche separation was estimated as between-species mean trait distances (C). Euclidean distances were calculated based on standardized trait data. Values are means across species ± 1 SE.

Averaged across species, niche width along single trait axes estimated as maximum value of all possible within-species trait distances (Fig. 1), was larger in mixture than in monoculture for leaf area (paired t-test: P < 0.05; Fig. 3A).

Niche width in other traits did not differ significantly between monoculture and mixture (paired t-test: P > 0.05). Niche density, quantified as within-species mean trait distances, was smaller in mixture than in monoculture in most traits related to height growth (shoot height, shoot length, internode length) and leaf morphology (leaf length, leaf area, maximum SLA) as well as in the positioning of shoot axes (stem angle), i.e. species were less tightly packed on these trait axes in mixture (Fig. 3B, S1 Table). Leaf positioning was the only trait where niche density was greater in the mixture than in monoculture. However, niche density in multiple traits did not differ between monocultures and the mixture (S1 Table). Additionally, niche density differed between species in all studied traits (S1 Table).

Niche separation (Fig. 1), estimated as mean of all possible pair-wise distances to other species per diversity-level (Fig. 3c, S1 Table), was larger in mixture than monoculture for leaf morphology (SLA, leaf area and leaf length). In contrast, niche separations along several trait axes related to space filling (stem and leaf angle) and height growth (shoot height and stretched length, SMF) were considerably greater among species in monoculture than in the mixture. Niche separation among species along multiple trait axes did not differ between monoculture and mixture (S1 Table).

**Fig 4:**
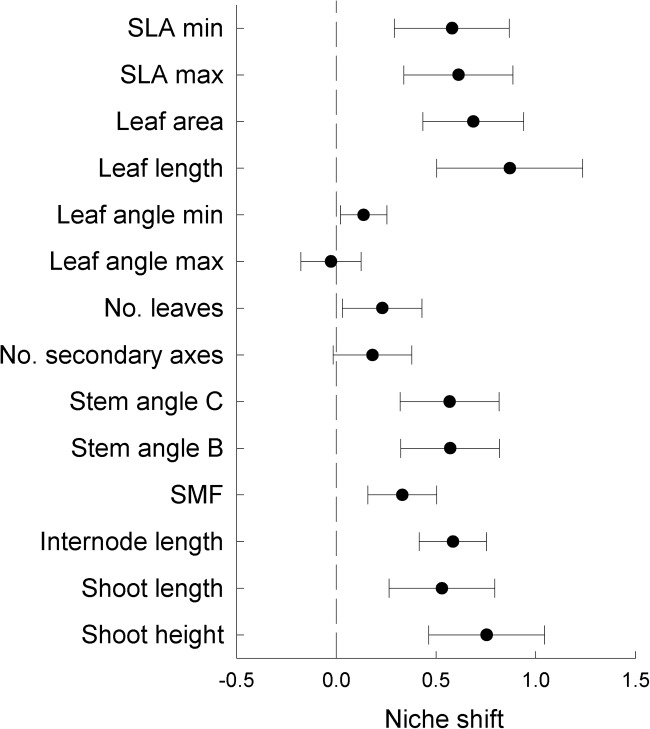
Niche shifts of legume species from monoculture to mixture. Niche shift was quantified as within-species trait distances of monoculture to mixture individuals and compared to within-species trait distances of monoculture individuals and is shown as log-response ratio. Euclidean distances were calculated based on standardized trait data. Values are means across species ± 1 SE. Positive values indicate that species shifted their niches in mixture compared to niche use in monoculture.

Niche shifts along trait axes (Fig. 1) quantified as mean distances between monoculture vs. mixture for each species was significant in 11 out of 14 traits (the three exceptions being maximum leaf angle, leaf number and number of secondary axes) from monoculture to mixture (Fig. 4, S1 Table). However, the degree of niche shift differed largely among species and from trait to trait (S1 Table).

### Total trait range of all legume species combined in monocultures and mixture

Total trait range estimated as maximum distances and filling of trait space estimated as mean distances across all individuals and species per diversity level were larger in mixture than in monoculture for leaf traits, i.e. morphology (specific leaf area, leaf area and length) and leaf number, as well as internode length (S3 Fig.). In contrast, filling of trait space was reduced in traits related to height growth (shoot height and length, stem mass fraction), stem and leaf positioning. In most cases, the reduced filling of trait space was associated with a reduction of total trait range. Using all traits together, total multivariate trait range was increased and total multivariate filling of trait space slightly decreased in mixture compared with monoculture, suggesting a less even distribution of species in the mixture.

### Relationships between diversity effects on species performance and trait-based niches

Performance in terms of shoot biomass was greater in the mixture than in monoculture in three studied legume species (*M*. *x varia*, *O*. *viciifolia*, *T*. *hybridum*; one-sample t-test: P < 0.05), while shoot biomass did not deviate from expected values in four legume species. Niche identity, i.e. trait values measured in the mixture, was significantly related to diversity effects on shoot biomass in all traits with exception of stem and leaf positioning (Table 3).

**Table 3:** Summary of mixed-effects models evaluating measures of trait-based niches as predictors for diversity effects on species performances in terms of shoot biomass.

Variable	Niche identity		Niche density		Niche separation		Niche shift	
	AIC	Chi^2^	AIC	Chi^2^	AIC	Chi^2^	AIC	Chi^2^
Multiple traits			88.94	2.50	90.03	1.41	91.43	0.01
Shoot height	23.47	67.97***↑	90.76	0.68	90.08	1.37	79.69	11.75***↑
Shoot length	6.95	84.49***↑	91.23	0.21	90.93	0.51	83.68	7.76**↑
Internode length	85.59	5.85*↑	89.17	1.73	91.10	0.34	91.29	0.15
Stem mass fraction	85.61	5.83*↑	84.70	6.74**↓	90.75	0.69	90.47	0.97
Stem angle basal	90.95	0.49	91.09	0.35	91.25	0.19	91.43	0.01
Stem angle canopy	91.06	0.39	91.09	0.35	91.25	0.19	91.43	0.01
No. secondary axes	33.49	57.95***↑	87.86	3.58	90.34	1.10	91.44	<0.01
Leaf number	-4.52	95.96***↑	88.83	2.61	91.33	0.11	91.43	0.01
Leaf angle max	87.20	4.24*↓	91.42	0.02	91.33	0.11	90.23	1.21
Leaf angle min	88.19	3.25	91.38	0.06	89.67	1.77	87.07	4.37*↑
Leaf length	53.89	37.55***↑	89.41	2.03	88.50	2.94	87.45	3.99*↓
Leaf area	31.87	59.57***↑	90.64	0.80	91.18	0.26	91.39	0.05
Specific leaf area max	71.78	16.66***↓	90.19	1.25	87.78	3.66	80.46	10.98***↓
Specific leaf area min	62.17	29.27***↓	89.97	1.47	86.64*↓	4.80*↓	85.48	5.96*↓

Alternative models were fitted with single predictor variables. Listed are AIC and Chi^2^ results of likelihood-ratio tests that were applied to assess model improvement and the statistical significance of the explanatory terms, where

* P ≤ 0.05,

** P < 0.01,

*** P < 0.001.

Arrows indicate positive (↑) or negative (↓) relationships of single predictors to diversity effects on shoot biomass.

Traits related to height growth (shoot height and length, internode length, SMF), space filling (number of secondary axes, leaf number) and leaf morphology (leaf area and length) were positively, while specific leaf area was negatively associated with increased shoot biomass in the mixture. Larger trait plasticity in response to plant diversity, i.e. niche shift from monoculture to the mixture, for shoot height and stretched length as well as leaf angle were positively related to increased performance in the mixture. In contrast, increased performance in the mixture was associated with smaller niche shifts in specific leaf area and leaf length (Table 3). Positioning in niche space in relation to other individuals of the same species (niche density) or other legume species (niche separation) did not show significant relationships with diversity-related changes in shoot biomass.

## Discussion

The need to consider both, inter- and intraspecific trait variation, is increasingly recognized in efforts to understand the mechanisms explaining species coexistence and community assembly [40,41]. Species interactions with the abiotic and biotic environment ultimately occur at the level of individuals. In diverse communities, different individuals of the same species are more likely to interact with individuals of different species than in less diverse communities. Recent studies in experimental grasslands showed remarkable differences in species means of plant functional traits in communities of different plant species diversity [31,42,43]. However, so far it is an open question whether increased plant diversity is related to higher intraspecific trait variation and whether diversity-related trait variation may increase niche partitioning among species.

### Trait variation in response to increased plant diversity

The niche concept of community ecology states that the realized niche of a species largely depends on the presence of other species [6]. In this concept species traits are basically assumed to be fixed such that the fundamental niche does not change. In our study, however, we could show that species traits can change in response to the presence of other species, thus in fact changing their fundamental niche at the same time as being pushed into the realized niche.

The extent of these diversity-induced changes in species traits differed largely between species and between traits (Fig. 2). Intraspecific trait variation may be constrained through genetic, environmental, developmental and stochastic factors [44]. In line with previous studies on functional trait responses to increased plant diversity and greater canopy density [31,42,43], plant height and stretched shoot length of legumes generally increased at higher plant diversity. Legume species with lowest height growth in the monoculture (*T*. *hybridum*, *T*. *pratense*, *V*. *cracca*), showed the strongest variation in size-related traits (increased shoot height, shoot length, internode length and SMF) and formed more erect shoot axes in the mixture than in monocultures. Increased height growth in smaller legume species in the mixture could not fully compensate growth differences to taller species (Fig. 2A), but plastic adjustment in other traits related to light acquisition was highly variable among legume species. For example, with exception of the tallest species (*M*. *x varia*, *O*. *viciifolia*) legume species could not reach the upper canopy levels in the mixture, but only two of them (*L*. *corniculatus*, *V*. *cracca*) showed a significant increase in SLA in the mixture, which is a well-known response to reduced light availability [20].

In general, it is likely that diversity-induced phenotypic plasticity accounted for a large amount of trait variation at the within-species level, although we cannot completely exclude that a diversity-mediated, short-term genetic or epigenetic differentiation had also occurred among monoculture and mixture populations of single species in the Jena Experiment [45]. Such changes could indeed be found after several years in the Jena Experiment [46], but in the present study we collected trait data on plants in the field established from the same seed source and cannot differentiate between these possibilities.

### Effects of plant diversity on phenotypic integration

Neighbouring plants cause substantial phenotypic responses in many plant species [47]. The extent of phenotypic plasticity may be limited by intrinsic or extrinsic factors [13,48]. Phenotypic integration, i.e. patterns and magnitude of character correlations [17], may play a role as internal constraint to intraspecific trait variation [15]. Phenotypic integration has been shown to increase with environmental stress and particularly where abiotic conditions are limiting [49,50], while other studies did not confirm this expectation [51]. In our study, the number of within-species trait correlations was higher in mixture than in monoculture, but the extraordinary tall-growing species *O*. *viciifolia* showed the lowest number of trait correlations both in monoculture and mixture (S2 Fig.). Higher community-level light interception at increased plant diversity [26] may increase competition for light, especially for subordinate species with a smaller growth stature which cannot be sufficiently compensated through phenotypic plasticity. This limitation is supported by the fact that our study could not include four other small-statured and short-lived legume species of the Jena Experiment species pool, i.e. *Medicago lupulina* L., *Trifolium campestre* Schreb., *T*. *dubium* Sibth. and *T*. *fragiferum* L., which were available in the monoculture but went extinct in the 60-species mixture.

### Niche overlap in mixture varies with trait dimensions

Niche complementarity is expected to be higher for phenotypically divergent species and individuals [44,46]. Based on the assumption, that complementarity plays a major role in more diverse plant communities we expected that niche separation among species should be larger, niche density within species should be greater and niche width within species should be smaller in mixture, thus lowering niche overlap among species in light acquisition traits compared with monocultures. However, it also has been proposed that individuals from different species are more similar in their trait values at increased plant diversity in order to minimize average fitness differences [52,53]. We found evidence for both: increased niche separation among legume species in the mixture for leaf traits (i.e. leaf length and area, SLA), but the opposite for traits related to height growth and positioning of stems and leaves (Fig. 3C, S1 Table). The co-occurrence of trait divergence and convergence may explain that diversity did not affect niche separation among legumes in multiple traits. Thus, multiple assembly processes observed in response to environmental variation in previous studies [54–57] also occur in communities of different plant species diversity.

In contrast to our expectation, niche width of individual species was not reduced and niche density of individual species was not greater in the mixture. Although a larger niche width and a smaller niche density of individual species increases the probability for niche overlap, it also may reduce niche overlap at the community level, if the total extent of occupied niche space is increased in a multi-species mixture [58]. Increasing within-species dissimilarities in height growth at increased species richness has also been reported from limestone grasslands and suggested to minimize competitive ability differences [59]. Accordingly, plasticity in height growth-related traits led to a convergence among species through enforced vertical foraging for light (increased apical dominance) in the mixture. Variation in leaf-morphological traits, however, suggested trait divergence in the mixture, thus increasing complementarity among species in light acquisition.

### Relationships between diversity effects on species performance and trait-based niches

In mixed plant communities, trait values determining a species`competitive ability (niche identity) as well as functional distinctiveness from other species (niche differences) are supposed to drive species performance and community assembly [52,53]. Greater values in traits related to height growth and space filling (Table 3) were important predictors for positive diversity effects on shoot biomass of legumes, emphasizing the important role of asymmetric competition for light in structuring grassland communities [60]. The greater similarity in traits related to height growth in the mixture and the positive effects of trait plasticity in shoot height and length (niche shift) on increased performance in the mixture supported the theory of equalizing fitness mechanisms [52]. In contrast, high SLA in the mixture and greater shifts in SLA from monoculture to the mixture was negatively related to species performance in the mixture. Obviously, trait variation in leaf morphology led to a greater exploitation of light at the multi-species level, but did not increase complementarity, i.e. increased the performance of all involved species.

Our study was based on low number of species and was restricted to monocultures and a high-diverse mixture. It is evident that further studies are needed to get a more general understanding to which extent intraspecific trait variation affects functional differences among species in communities of varying plant diversity and to further develop a theoretical framework how measurable traits may be used to quantify niche differences among species. However, our study showed that increased trait-based niche differences among species and increased total niche width of multiple species in mixtures are limited to traits that are not related to asymmetric competition for light and height growth, but the interplay of multiple niche dimensions and different plasticities of species along these niche dimensions may increase total resource use in more diverse communities. Functional equivalence, i.e. the convergence in traits related to height growth, seems to be an important mechanism explaining the positive effects of plant diversity on individual species in our experimental grasslands. Our study clearly shows that trait-based analyses in order to explain species coexistence and biodiversity require the incorporation of intraspecific trait variation.

## Supporting Information

S1 Fig Reaction norm of traits to increased plant diversity. (DOC)

S2 Fig Correlation structure among traits in monoculture and the mixture for each legume species. (DOC)

S3 Fig Filling of trait space (mean distances) and total trait range (maximum distances) comparing mixture vs. monoculture. (DOC)

S1 Table Summary of statistical analyses for niche density, niche separation and niche shift. (DOC)
