# Supplementary material for: Contrasting Effects of Intraspecific Trait Variation on Trait-Based Niches and Performance of Legumes in Plant Mixtures
Source: PLoS One. 2015 Mar 17;10(3):e0119786. doi: 10.1371/journal.pone.0119786 (PMC4363318; doi:10.1371/journal.pone.0119786)
Supplement: S1 Fig — (DOC) [file pone.0119786.s001.doc]

**S1 Figure. Reaction norm of traits to increased plant diversity.** Variation in single traits is expressed as regression slopes of standardized trait values against plant diversity (monoculture vs. mixture) for each studied legume species.
