# Supplementary material for: Contrasting Effects of Intraspecific Trait Variation on Trait-Based Niches and Performance of Legumes in Plant Mixtures
Source: PLoS One. 2015 Mar 17;10(3):e0119786. doi: 10.1371/journal.pone.0119786 (PMC4363318; doi:10.1371/journal.pone.0119786)
Supplement: S2 Fig — (DOC) [file pone.0119786.s002.doc]

**S2 Figure. Correlation structure among traits in monoculture and the mixture for each legume species.** Left panels show relationships in monoculture, right panels show relationships in the mixture for (A, B) *Lathyrus pratensis*, (C, D) *Lotus corniculatus*, (E, F) *Medicago x varia*, (G, H) *Onobrychis viciifolia*, (I, K) *Trifolium hybridum*, (L, M) *T. pratense*, and (N, O) *Vicia cracca*. Solid lines indicate positive correlations, dashed lines show negative correlations.

Abbreviations of variable names: S-Height = shoot height, S-Length = shoot length, S-Ang_B_ = stem angle basal, S-Ang_C_ = stem angle upper shoot part, L_int_ = internode length, SMF = stem mass fraction, #Axes = number of secondary axes, #Leaf = number of leaves, L-Ang_max_ = maximum leaf angle, L-Ang_min_ = minimum leaf angle, L-Length = leaf length, L-Area = leaf area, SLA_max_ = maximum specific leaf area, SLA_min_ = minimum specific leaf area.

**continued S2 Figure**
