# Supplementary material for: Contrasting Effects of Intraspecific Trait Variation on Trait-Based Niches and Performance of Legumes in Plant Mixtures
Source: PLoS One. 2015 Mar 17;10(3):e0119786. doi: 10.1371/journal.pone.0119786 (PMC4363318; doi:10.1371/journal.pone.0119786)
Supplement: S3 Fig — (DOC) [file pone.0119786.s003.doc]

**S3 Figure. Filling of trait space (mean distances) and total trait range (maximum distances) comparing mixture vs. monoculture.** Shown are log response ratios (lnRR) between the mixture and the monoculture. Positive values indicate that filling of trait space was less tight and total trait range was larger in the mixture than in the monoculture, while negative values indicate the opposite.
