# Supplementary material for: Contrasting Effects of Intraspecific Trait Variation on Trait-Based Niches and Performance of Legumes in Plant Mixtures
Source: PLoS One. 2015 Mar 17;10(3):e0119786. doi: 10.1371/journal.pone.0119786 (PMC4363318; doi:10.1371/journal.pone.0119786)
Supplement: S1 Table — (DOC) [file pone.0119786.s004.doc]

**S1 Table. Summary of statistical analyses for niche density, niche separation and niche shift.** Analysis of variance (ANOVA) was applied to test for effects of legume species identity and plant diversity on niche density (= within-species mean trait distances, i.e. greater distances indicate lower niche density), niche separation (= between-species mean trait distances) and niche shift (= trait distances between all monoculture individuals per species compared to trait distances between monoculture and mixture individuals) in traits related to light acquisition

| **Niche density** | Species | Diversity | Species x Div | Lp | Lc | Mv | Ov | Th | Tp | Vc |
| --- | --- | --- | --- | --- | --- | --- | --- | --- | --- | --- |
| all traits | 18.54*** | 0.64 | 0.57 | -- | -- | -- | -- | -- | -- | -- |
| Shoot height | 28.93*** | 100.57***↑ | 20.95*** | ***↑ | -- | ***↑ | *↓ | ***↑ | ***↑ | ***↑ |
| Shoot length | 45.28*** | 26.30***↑ | 21.00*** | -- | -- | ***↑ | *↓ | ***↑ | ***↑ | -- |
| Internode length | 9.26*** | 11.23**↑ | 12.33*** | ***↑ | -- | -- | -- | -- | **↓ | -- |
| Stem mass fraction | 9.33*** | 0.27 | 7.23*** | ***↑ | -- | -- | -- | -- | -- | *↓ |
| Stem angle basal | 14.58*** | 8.43**↑ | 11.25*** | -- | -- | -- | ***↑ | -- | -- | -- |
| Stem angle canopy | 14.15*** | 8.44**↑ | 11.51*** | -- | -- | -- | ***↓ | -- | -- | -- |
| No. secondary axes | 13.95*** | 1.53 | 5.69*** | -- | -- | -- | -- | *↓ | -- | -- |
| Leaf number | 24.77*** | 3.28 | 15.18*** | -- | -- | -- | ***↓ | -- | ***↑ | -- |
| Leaf angle max | 37.39*** | 17.47***↓ | 17.43*** | -- | -- | ***↓ | -- | -- | ***↓ | ***↓ |
| Leaf angle min | 21.24*** | 47.72***↓ | 17.19*** | -- | -- | -- | ***↓ | -- | -- | -- |
| Leaf length | 30.79*** | 128.59***↑ | 25.99*** | *↑ | ***↑ | -- | -- | ***↑ | -- | -- |
| Leaf area | 20.34*** | 108.90***↑ | 25.16*** | ***↑ | ***↑ | ***↑ | ***↓ | ***↑ | -- | -- |
| Specific leaf area max | 9.43*** | 4.01*↑ | 9.77*** | ***↓ | -- | -- | ***↑ | *↑ | -- | -- |
| Specific leaf area min | 10.64*** | 0.22 | 8.68*** | *↓ | -- | -- | -- | -- | -- | ***↑ |
|  |  |  |  |  |  |  |  |  |  |  |
| **Niche separation** | Species | Diversity | Species x Div | Lp | Lc | Mv | Ov | Th | Tp | Vc |
| all traits | 11.32*** | 3.52 | 3.08** | -- | -- | -- | -- | -- | -- | -- |
| Shoot height | 21.19*** | 38.31***↓ | 4.20*** | -- | -- | ***↓ | -- | -- | *↓ | **↓ |
| Shoot length | 18.71*** | 45.86***↓ | 4.77*** | -- | -- | ***↓ | -- | -- | **↓ | -- |
| Internode length | 8.68*** | 3.45 | 0.90 | -- | -- | -- | -- | -- | -- | -- |
| Stem mass fraction | 3.49** | 81.61***↓ | 2.99** | -- | ***↓ | **↓ | -- | ***↓ | -- | -- |
| Stem angle basal | 2.52* | 19.45***↓ | 3.12** | -- | -- | -- | -- | -- | -- | ***↓ |
| Stem angle canopy | 1.46 | 27.72***↓ | 1.91 | -- | -- | -- | -- | -- | -- | ***↓ |
| No. secondary axes | 11.59*** | <0.01 | 2.85* | -- | -- | -- | -- | -- | -- | -- |
| Leaf number | 19.07*** | <0.01 | 2.23* | -- | -- | -- | -- | -- | -- | -- |
| Leaf angle max | 1.23 | 17.77***↓ | 0.95 | -- | -- | -- | -- | -- | -- | -- |
| Leaf angle min | 0.85 | 72.31***↓ | 0.53 | *↓ | -- | *↓ | *↓ | **↓ | -- | -- |
| Leaf length | 44.88*** | 11.10**↑ | 11.21*** | -- | -- | -- | -- | -- | ***↓ | -- |
| Leaf area | 26.56*** | 28.60***↑ | 3.31** | -- | -- | -- | -- | -- | -- | -- |
| Specific leaf area max | 6.14*** | 42.25***↑ | 4.84*** | -- | *↑ | -- | ***↑ | -- | -- | -- |
| Specific leaf area min | 10.57*** | 105.97***↑ | 3.94** | *↓ | ***↓ | -- | ***↓ | *↓ | -- | -- |
|  |  |  |  |  |  |  |  |  |  |  |
| **Niche shift** | Species | Diversity | Species x Div | Lp | Lc | Mv | Ov | Th | Tp | Vc |
| all traits | 23.81*** | 91.24*** | 5.48*** | -- | -- | ** | -- | ** | *** | *** |
| Shoot height | 30.97*** | 239.46*** | 32.76*** | *** | -- | -- | -- | *** | *** | *** |
| Shoot length | 45.97*** | 98.66*** | 21.98*** | -- | -- | ** | -- | *** | *** | -- |
| Internode length | 26.18*** | 95.72*** | 7.55*** | *** | *** | *** | -- | ** | ** | -- |
| Stem mass fraction | 18.37*** | 31.88*** | 7.80*** | ** | -- | -- | -- | *** | -- | -- |
| Stem angle basal | 11.41*** | 39.84*** | 8.43*** | -- | -- | -- | *** | -- | -- | *** |
| Stem angle canopy | 10.96*** | 39.67*** | 8.45*** | -- | -- | -- | *** | -- | -- | *** |
| No. secondary axes | 10.32*** | 2.05 | 5.08*** | -- | -- | -- | -- | -- | ** | -- |
| Leaf number | 20.14*** | 2.03 | 4.67*** | -- | -- | -- | -- | -- | * | -- |
| Leaf angle max | 16.92*** | 0.03 | 2.82* | -- | -- | -- | -- | -- | -- | -- |
| Leaf angle min | 4.84*** | 6.21* | 1.67 | -- | -- | -- | -- | -- | -- | -- |
| Leaf length | 15.85*** | 167.23*** | 22.86*** | -- | *** | *** | -- | *** | ** | -- |
| Leaf area | 16.67*** | 96.13*** | 12.32*** | *** | *** | *** | -- | * | ** | -- |
| Specific leaf area max | 7.54*** | 78.96*** | 15.03*** | -- | -- | -- | *** | * | -- | *** |
| Specific leaf area min | 7.87*** | 59.80*** | 15.61*** | -- | -- | -- | *** | * | -- | *** |

The first columns show F ratios related to the tested factors (Species = legume species identity, 7 factor levels; Diversity = monoculture vs. mixture, 2 factor levels) and levels of significance, where * P ≤ 0.05, ** P < 0.01, *** P < 0.001. Arrows indicate increases (↑) or decreases (↓) in mixture compared to monocultures. Tukey`s test was applied for multiple pair-wise comparisons of sample means per species. Abbreviations are Lp = *Lathyrus pratensis*, Lc = *Lotus corniculatus*, Mv = *Medicago x varia*, Ov = *Onobrychis viciifolia*, Th = *Trifolium hybridum*, Tp = *Trifolium pratense*, Vc = *Vicia cracca*.
